# Therapeutic potential of bacteriophage endolysins for infections caused by Gram-positive bacteria

**DOI:** 10.1186/s12929-023-00919-1

**Published:** 2023-04-26

**Authors:** He Liu, Zhen Hu, Mengyang Li, Yi Yang, Shuguang Lu, Xiancai Rao

**Affiliations:** 1 Department of Microbiology, College of Basic Medical Sciences, Key Laboratory of Microbial Engineering Under the Educational Committee in Chongqing, Army Medical University, Chongqing, 400038 China; 2 Department of Microbiology, School of Medicine, Chongqing University, Chongqing, 400044 China

**Keywords:** Bacteriophage, Endolysins, Gram-positive bacteria, Infectious diseases

## Abstract

Gram-positive (G^+^) bacterial infection is a great burden to both healthcare and community medical resources. As a result of the increasing prevalence of multidrug-resistant G^+^ bacteria such as methicillin-resistant *Staphylococcus* *aureus* (MRSA), novel antimicrobial agents must urgently be developed for the treatment of infections caused by G^+^ bacteria. Endolysins are bacteriophage (phage)-encoded enzymes that can specifically hydrolyze the bacterial cell wall and quickly kill bacteria. Bacterial resistance to endolysins is low. Therefore, endolysins are considered promising alternatives for solving the mounting resistance problem. In this review, endolysins derived from phages targeting G^+^ bacteria were classified based on their structural characteristics. The active mechanisms, efficacy, and advantages of endolysins as antibacterial drug candidates were summarized. Moreover, the remarkable potential of phage endolysins in the treatment of G^+^ bacterial infections was described. In addition, the safety of endolysins, challenges, and possible solutions were addressed. Notwithstanding the limitations of endolysins, the trends in development indicate that endolysin-based drugs will be approved in the near future. Overall, this review presents crucial information of the current progress involving endolysins as potential therapeutic agents, and it provides a guideline for biomaterial researchers who are devoting themselves to fighting against bacterial infections.

## Introduction

The extensive use of antibiotics promotes the crisis of antimicrobial resistance (AMR), which has made the clinical treatment of bacterial infections difficult and poses a challenge to global public health; the AMR problem requires immediate action, preferably one that is long term [1, 2]. Drug-resistant bacterial infections can result in at least 50,000 deaths every year in Europe and the United States and hundreds of thousands of victims in other regions of the world [3], leading to a loss of $3 trillion in gross domestic product annually [4]. In 2017, the World Health Organization published a list of global priority pathogens that require the exploration and development of novel antimicrobials [5]. Among these pathogens, Gram-positive (G^+^) bacteria occupy a large proportion in the clinical detection of drug-resistant bacteria, especially methicillin-resistant *Staphylococcus* *aureus* (MRSA), vancomycin-resistant *Enterococcus* *faecium*, and β-lactamase-resistant *Streptococcus* *pneumoniae,* which are major healthcare problems [5]. Therefore, novel antimicrobial agents must be urgently developed to combat the infections caused by drug-resistant G^+^ bacteria.

**Fig. 1:**
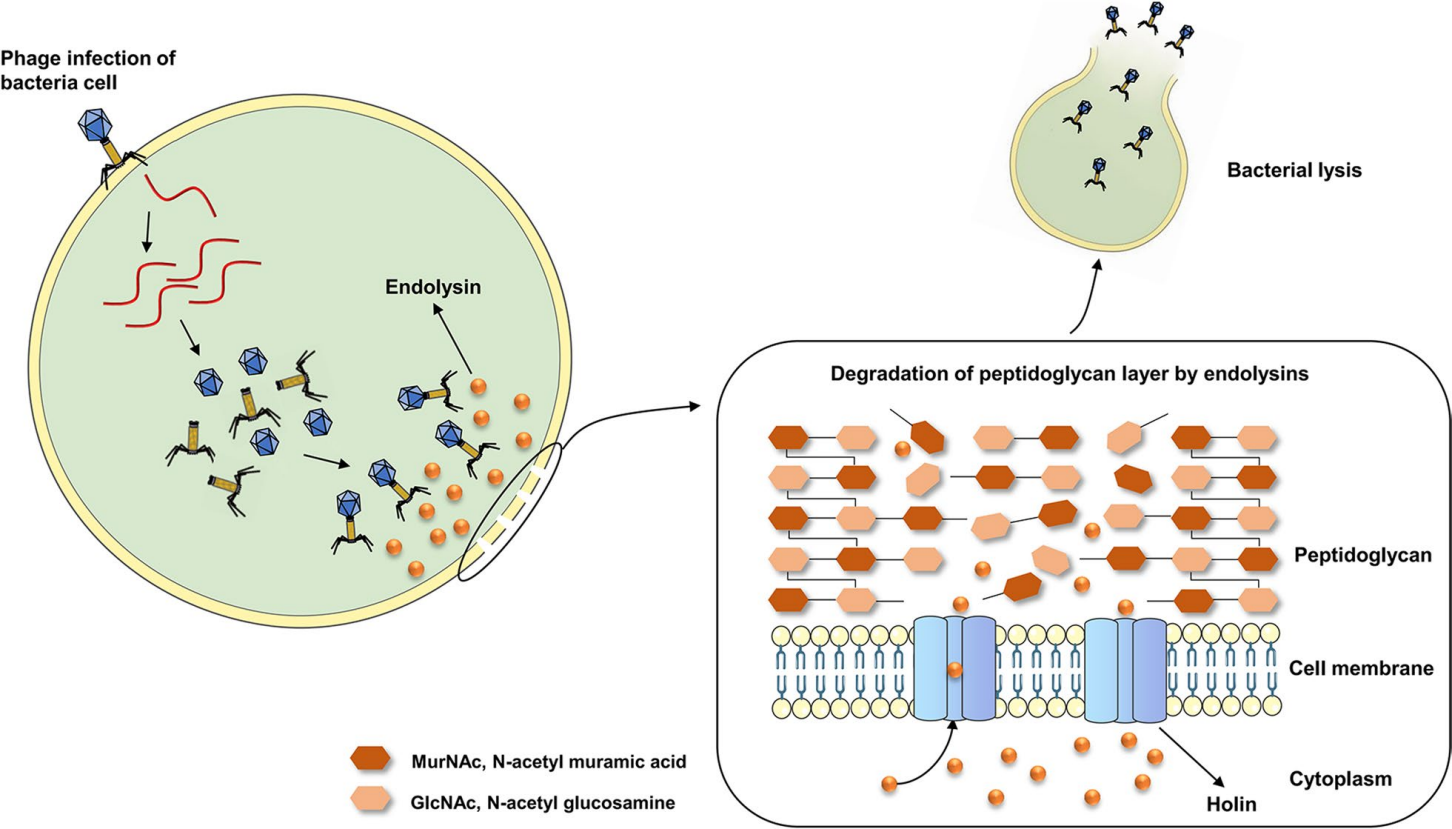
The role of endolysins and holins in the process of phage infection of a G^+^ bacterium. After replication inside the bacterial cell, progeny phages utilize a lytic system including endolysins and holins to destroy the integrity of the cell wall from the inside of the bacterium and release the assembled phage virions

Bacteriophages (phages) are the most abundant biological entities on earth. They are widespread all over the biosphere from the soil to marine environments, the atmosphere, and the human body. Phages are viruses that can specifically infect and rapidly kill the bacterial hosts in their lytic life cycles [6, 7]. After replication inside the bacterial cells, phages need to exit from the bacterial hosts to release assembled progeny virions. The phages evolved a lytic system to digest the bacterial cell wall, thereby inducing bacterial lysis [8]. Phage endolysins are highly efficient molecules that have been used by phages for billions of years for this exact purpose. Endolysins can access the peptidoglycan through membrane lesions formed by the second phage-encoded proteins (holins); they degrade the integrity of the cell wall from the inside of the bacteria (Fig. 1) [9, 10]. About half of the bacteria on earth can be killed by their phages in 48 h, making endolysins the most effective and widespread bactericidal agents on the planet [7]. Although intact phages can also be an antibacterial option, endolysins have more advantages compared with phage particles, making them important candidates for use as alternatives to antibiotics [11, 12].

The presence of the outer membrane of Gram-negative (G^−^) bacteria effectively presents a physical protective barrier against endolysins, which can directly target the bonds in the peptidoglycan and lyse the cell wall of G^+^ bacteria that do not have outer membranes [13–15]. This discovery prompted scientists to attempt to harness the bacteriolytic properties of endolysins to treat G^+^ bacterial infections. Many recombinant endolysins have already been expressed, identified, and purified; they sufficiently display potent bacteriolytic activity against G^+^ bacteria [15–17]. In addition, phage endolysins can eradicate staphylococcal and streptococcal biofilms in a short time [18–20]. For example, CF-301 removes all biofilms in catheters within 1 h [20], purified CHAP_K_ completely eliminates the staphylococcal biofilms within 4 h [21], and ClyF decreases the 25.2–93.5% biofilm mass within 45 min [22]. Furthermore, multiple in vitro and in vivo experiments have demonstrated that endolysins, such as PlyC [23], PlyG [24], Cpl-1 [25], CHAP_K_ [18], LysGH15 [26], and LysP108 [11], are effective against a variety of G^+^ bacterial infections. The endolysin-based candidate drugs such as P128 and N-Rephasin® SAL200 are being tested in phases II and IIa in the treatment of *S.* *aureus* bacteremia, respectively [27–29]. However, CF-301 failed in the phase III clinic trials. Here, we present an overview of the characteristics and antimicrobial potential of endolysins derived from phages and evaluate whether they can alternate or sensitize conventional antibiotics in the treatment of G^+^ bacterial infections.

### Structure and classification of endolysins

**Fig. 2:**
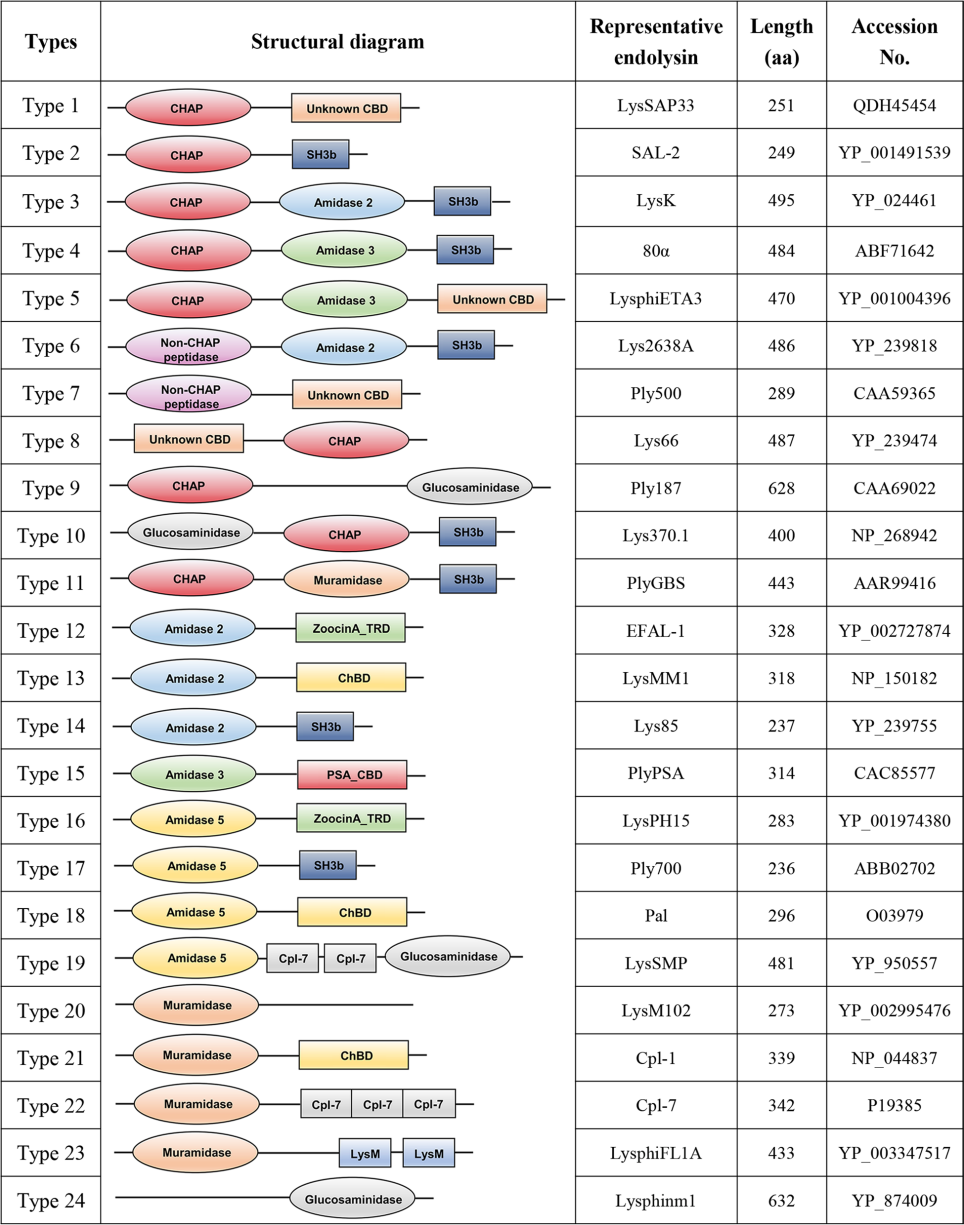
The typical modular structures of different types of endolysins derived from phages targeting G^+^ bacteria. 24 types of endolysins are proposed according to their molecule structures. *CHAP* cysteine- and histidine-dependent aminopeptidase/hydrolase, *SH3* bacterial Src homology 3 domain, responsible for cell-wall peptidoglycan recognition and binding, *ChBD* choline-binding domain, *PSA_CBD* cell wall-binding domain, *ZoocinA_TRD* a target recognition domain, *Cpl-7* Cpl-7-like cell wall-binding domain, *LysM* a small domain involved in binding peptidoglycan

The structures of phage endolysins are determined by their origin. In General, endolysins produced by phages infecting G^−^ bacteria (molecular weight, 15–20 kDa) have a simple globular configuration, whereas most of the endolysins derived from phages targeting G^+^ bacteria (molecular weight, 25–40 kDa) comprise two modular structures: an N-terminal catalytic domain (CD) joined by a flexible linker to a C-terminal cell wall-binding domain (CBD) [30–32]. Some of them feature a modular architecture comprising two different types of functional domains linked jointly to a single CBD, particularly staphylococcal endolysins [33, 34]. A central CBD can separate the two CDs, and this structure is presented in streptococcal endolysins λSA2 and PlySK1249 [35–37]. Specifically, a unique endolysin Ply187, from a *S.* *aureus* phage 187, has two CDs but lacks a CBD [38–40]. Therefore, the abundant modular structures of endolysins are diverse, and the function of different CDs and CBDs is distinct. To better understand these complex endolysins derived from phages targeting G^+^ bacteria (mainly including *Staphylococcus*, *Streptococcus*, *Enterococcus,* and *Listeria*), we propose a systematic classification of these endolysins based on their domain compositions (Fig. 2) and update other types of staphylococcal endolysins given that they were classified into six types [41, 42]. The information from the National Center for Biotechnology Information database on the representatives of different types of endolysins is shown in Fig. 2. In general, the N-terminal CD of endolysin is responsible for hydrolyzing various specific peptidoglycan bonds of G^+^ bacteria [14, 43, 44]. By contrast, the C-terminal CBD recognizes and non-covalently binds to different ligands (usually carbohydrate) in the cell wall for proper fixation of the CDs [13, 45, 46]. Although the C-terminal CBD is required to maintain the intact lytic activity of CDs [47, 48], truncation or deletion of the CBD can also result in equal or increased lytic activity of the mutants [43, 49, 50].

Sequence comparison of endolysins of the same type shows high homology within the N-terminal enzymatically active domain and low similarity within the C-terminal cell binding region [14, 51]. The phages that infect G^+^ bacteria have naturally designed such distinct domain structures to better disseminate the progeny particles [8]. The similarity of the amino acid sequences of the endolysin CDs may be explained by the conserved peptidoglycan bonds of bacterial hosts, whereas most of the CBDs may have evolved to target unique components of the cell wall of the host bacteria at high affinity, thereby resulting in variability, high selectivity, and low propensity for developing resistance [15, 51, 52]. The modular structure of endolysins can be exploited for bioengineering, because different domains can be genetically swapped or shuffled among different endolysins, thereby generating novel fused enzymes with high specificity and catalytic activity [52–54]. For example, the recombinant chimeric endolysin PRF-119, which was designed with a CD, a cysteine- and histidine-dependent aminopeptidase/hydrolase (CHAP) domain from the endolysin of phage K, and a CBD from the lysostaphin, is highly active against *S.* *aureus*, including MRSA [55]. In addition, as a chimeric phage endolysin, Ply187AN-KSH3b exhibits strong antimicrobial activity against *S.* *aureus*, including disruption of biofilms and protection of mice from *S.* *aureus* endophthalmitis [56]. Therefore, the modular arrangement of endolysins has enormous potential in the creative design of important enzymes with specific functions or features.

### Mode of endolysin action

**Fig. 3:**
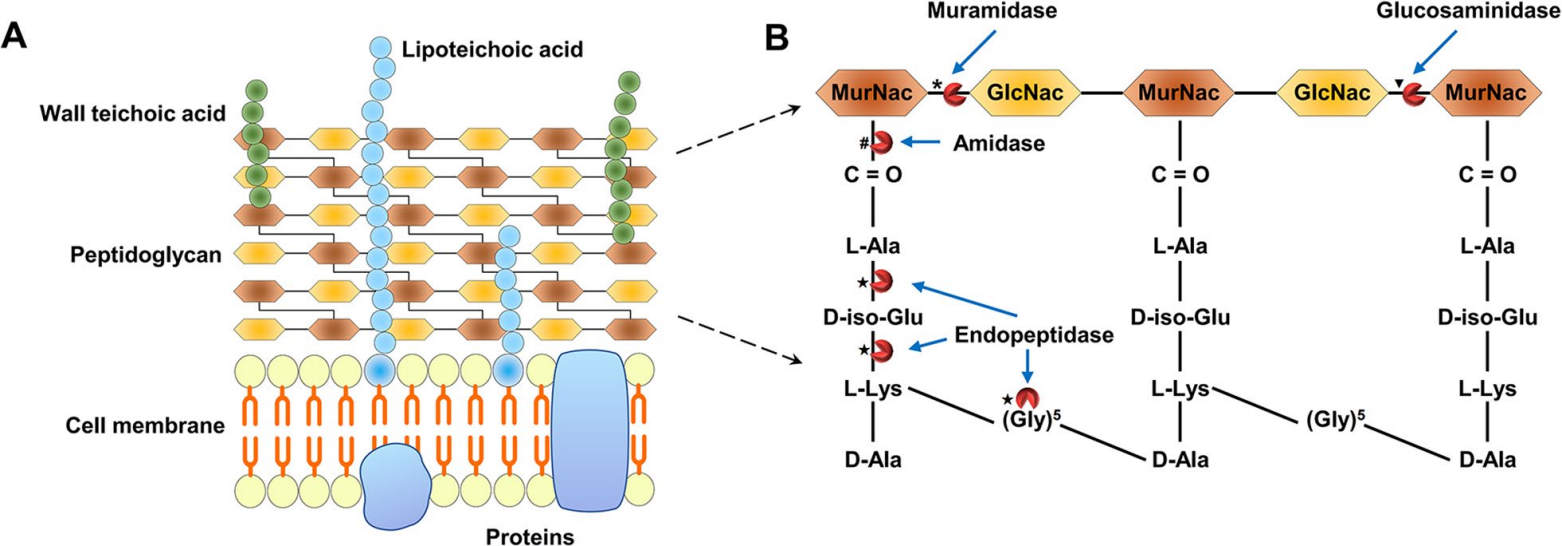
The function of endolysin catalytic domains encoded by phages infecting G^+^ bacteria. **A** Schematic representation of the G^+^ bacterial cell wall. **B** Diagram of the peptidoglycan bonds cleaved by different endolysins. MurNAc and GlcNAc are repeating units of the glycan strands that are linked to a stem peptide through an amide bond to the MurNAc. Stem peptides are then cross-linked through a pentaglycine (in the case of *S.* *aureus*) to adjacent stem peptides forming a tight stable net around the bacterium. Based on the cleaved chemical bonds within the peptidoglycan layer, endolysins have several enzyme activities, including muramidase (N-acetylmuramidase), glucosaminidase (N-acetyl-β-D-glucosaminidases), amidase (N-acetylmuramoyl-L-alanine amidase), and endopeptidase (L-alanoyl-D-glutamate endopeptidase or interpeptide bridge-specific endopeptidases). *MurNAc* N-acetyl muramic acid, *GlcNAc* N-acetyl glucosamine, *L-Ala* L-alanine, *D-iso-Glu* D-iso-glutamic acid, *L-Lys* L-lysine, *D-Ala* D-alanine. *β-1,4 glycosidic bond between MurNAc and GlcNAc. ▼β-1,4 glycosidic bond between GlcNAc and MurNAc. **#**amide bond between MurNAc and L-Ala. ★peptide bond between two amino acids

The modular structure of endolysins is closely related to their mode of action. With the individual binding specificity of CBD, endolysin CDs kill bacteria by enzymatically degrading the peptidoglycan of the bacterial cell wall, which protects the cell protoplast from mechanical damage and osmotic lysis and is essential to bacterial viability. Compared with G^−^ bacteria, the cell walls of G^+^ bacteria are thicker (15–80 nm) and consist of tens of layers of peptidoglycan associated with teichoic acids (Fig. 3A) [51]. Apart from lytic transglycosylases (e.g., phage λ lysozyme), endolysins are peptidoglycan hydrolases that use a water molecule to catalyze the cleavage of different bonds (Fig. 3B), such as β-1,4 glycosidic bond, amide bond, and peptide bond [51]. Most staphylococcal phage endolysins have two catalytic domains: a CHAP domain with D-Ala-Gly activity and an amidase domain with MurNAc-L-Ala activity [57]. Electron microscopy revealed that endolysin-mediated peptidoglycan digestion leads to perforation in the cell wall, through which the high intracellular osmotic pressure squeezes the cytoplasmic membrane to cause hypotonic lysis of the bacteria within seconds. By contrast, antibiotics depend on the inhibition of a metabolic pathway and require more steps and time to arrest bacterial growth or kill bacterial cells [14, 16, 52, 58]. Moreover, endolysins can effectively eliminate staphylococcal biofilms and reduce bacterial persisters due to the active mode of action, resulting in the successful therapy of chronic infections after treatment failure by antibiotics [59, 60].

### Efficacy of endolysins

Endolysins show bactericidal activity against certain bacterial species that are closely related to the bacterial hosts of the phages from which they were produced. Despite their strong specificity, the host range of endolysins can reach approximately two-thirds of the tested strains, and some even reach 100% (Table 1), which is significantly stronger than the host range of the phage itself [36]. For instance, a purified pneumococcal phage endolysin (Pal) can kill 15 common serotypes of pneumococci, including highly penicillin-resistant strains [16]. In many cases, endolysins might be identified with extended lytic activity (Table1). For instance, LysPBC2 was isolated from a *Bacillus* *cereus* phage and displayed very broad lytic activity against all *Bacillus*, *Listeria*, and *Clostridium* species tested [70]. An enterococcal phage endolysin PlyV12 reportedly kills not only enterococci but also several other G^+^ pathogens, such as streptococci and staphylococci [71]. Furthermore, endolysins have been successfully exploited to kill G^+^ pathogenic bacteria in a dose-dependent manner, including antibiotic-sensitive bacteria and antibiotic-resistant ones, such as *B.* *anthracis* and *B.* *cereus* [24], *C.* *difficile* [71], *C.* *perfringens* [72], *E.* *faecalis* and *E*. *faecium* [70], *L.* *monocytogenes* [58], *S.* *aureus* [73], *S.* *agalactiae* [74, 75], and *S.* *pyogenes* [76]. An earlier study found that 2 units (U) (2 μg) of recombinant phage endolysin PlyG can destroy 1.0 × 10^4^ colony formation unit (CFU) of streptomycin-resistant *B.* *cereus* within 10 s [24]. In a separate kinetic assay, the addition of 2 U of PlyG to 1 mL of log-phase *B.* *cereus* cells resulted in a 17,000-fold decrease of bacterial numbers within 20 s and near sterilization at 2 min when compared with 50 mM Tris buffer treatment [24]. Therefore, endolysin has strong lytic efficacy against bacterial cells.

**Tabel 1:** The host range of representative endolysins

Endolysin and/or derivative	Origin	The number of tested strains	The number of lysed strains	Host range	References
SAL200	Staphylococcal phage SAP-1	425	425	100%	[61]
Exebacase (CF-301 or PlySs2)	Prophage of *Streptococcus* *suis*	477	365	77%	[62]
P128	CHAP domain (TAME phage K) + SH3b (lysostaphin)	62	62	100%	[63]
Staphefekt SA.100	M23 endopeptidase (lysostaphin) + Amidase (Ply2638) + SH3b (Ply2638)	11	10	91%	[64]
XZ.700	Staphefekt SA.100 deleted 44 amino acids region	120	107	89%	[64]
LysK	Staphylococcal phage K	27	18	67%	[65]
CHAP_K_ (truncated LysK)	Staphylococcal phage K	31	28	90%	[66]
ClyF	CD domain (Ply187) + CBD domain (PlySs2)	51	45	88%	[22]
LysGH15	Staphylococcal phage GH15	57	52	91%	[67]
LysH5	Staphylococcal phage PhiH5	90	77	86%	[68]
Pal	Streptococcal phage Dp-1	25	19	76%	[16]
Cpl-1	Streptococcal phage Cp-1	27	22	81%	[69]
PlyG	*B.* *anthracis* γ-phage	27	16	59%	[24]

*TAME* tail-associated muralytic enzymes, *CHAP* cysteine- and histidine-dependent aminopeptidase/hydrolase, *CD* catalytic domain, *CBD* cell wall-binding domain

Unlike antibiotics, which are small molecules and generally non-immunogenic, one of the potential concerns with endolysin treatment is the adverse immune response induced by the generation of neutralizing antibodies that may reduce in vivo endolysin activity after systemic and mucosal application [8, 77]. Early studies have confirmed that although endolysins are immunogenic, antibodies against the corresponding endolysins specific for *B.* *anthracis*, *S.* *aureus*, *S*. *pneumoniae,* or *S.* *pyogenes,* obtained from rabbit hyperimmune serum do not remarkably diminish lytic activity in vitro [76, 78, 79]. For example, the bactericidal activity and binding capacity of staphylococcal-specific endolysin LysGH15 were not blocked even after incubation with anti-LysGH15-serum for 60 min [26]. Furthermore, experiments with pneumococcal-specific endolysin Cpl-1 in immunized rabbit serum (in vitro) and immunized mice (in vivo) did not affect its therapeutic efficacy [69]. These results were verified with endolysins MV-L and Pal [80, 81]. Collectively, endolysins are hardly affected by the immune response. Thus, they have almost no loss of efficacy or adverse effect when applied to treat bacterial infections, which may be partially explained by the strong binding affinity of an endolysin to its cell wall substrate and rapid bactericidal activity, which outcompetes the hosts’ immune response [8, 79].

### Advantages of endolysins used as antimicrobial agents

Given the resistance crisis, phage therapy was proposed and served as a powerful regimen for clinical infections [36]. However, the narrow antimicrobial spectrum, complicated pre-clinical and clinical evaluation, and improper regulatory framework of phages hamper the wide application of phage therapy [27, 36]. Compared with active phages, endolysins develop considerably faster and have many advantages, such as non-proliferation, fast bactericidal activity, wide host spectrum, definite pharmacokinetics, and low possibility of resistance development (Table 2), making endolysins important candidates as the alternatives of antibiotics, especially for drug-resistant bacteria. Among these advantages, low possibility of resistance development is most prominent for endolysins to overwhelm phage therapy and antibiotics [78]. Phages have coevolved with bacteria for billions of years. To avoid being trapped in the host, the CBD of endolysins has evolved to target the highly conserved bonds within the peptidoglycan of the cell wall, which is necessary for bacterial viability; thus, resistance to these enzymes is a rare event [8, 52]. This speculation was confirmed by the evidence that the binding epitopes for endolysin CBD in the cell walls of pneumococci, Group A streptococci, and *B.* *anthracis* are choline [91], polyrhamnose [84], and neutral polysaccharide [24], respectively, which are important molecules for bacterial growth. To our knowledge, no case of resistance to endolysins has ever been reported; thus, corresponding mutants hardly survive. Even repeated exposure of staphylococci, pneumococci, and *B*. *cereus* to low concentrations of endolysins on agar plates or in broth culture does not identify spontaneously resistant mutants, whereas a concomitant 1,000-fold and 10,000-fold increase in novobiocin and streptomycin resistance could be observed [16, 24]. Endolysin ClyS displays a decreased potential for the development of resistance compared with mupirocin when MRSA or methicillin-sensitive *S.* *aureus* (MSSA) was exposed to increasing concentrations (1/32 × to 4 × minimal inhibitory concentration, MIC) of either agent for over 8 days in vitro [78]. LysGH15 also does not induce resistance in MRSA or MSSA strains after repeated treatment with sub-MIC [26]. The expression of thick polysaccharide capsules by streptococci or *B.* *anthracis* or the formation of dense biofilms by staphylococci or streptococci does not block endolysin lytic activity [74, 79, 92]. Therefore, the intrinsic endolysin resistance is seldom, which is a great advantage for the use of endolysin as a promising therapeutic agent.

**Table 2:** Comparison of the properties of antibiotic, phage, and endolysin as antibacterial therapeutic agents

Properties	Antibiotic	Phage	Endolysin	References
Bacteriocidal specificity	Broad spectrum more common than narrow spectrum	Typically narrow, species or strain specificity	Relatively broad lytic activity	[82, 83]
Proliferation	Non-proliferation	Self-proliferation	Non-proliferation	[11, 84]
Mode of action	Applied from without, target specific sites, typically disrupts one bacterial process	Applied from without, disrupt many essential cellular processes	Applied from without, target bonds in the peptidoglycan	[83, 85]
Bacteriocidal speed	Short time between administration and eradication of bacteria	Long time between administration and eradication of bacteria	Rapid bacterial activity within seconds of contact	[14, 86]
Intracellular activity	Diffusion through membranes allows for treatment of intracellular bacteria	Unable to penetrate eukaryotic cells	Few or modified ones (e.g., CPP-fused endolysins) can enhance intracellular efficacy	[77, 86, 87]
Resistance development	Prone to develop resistance	Resistance occurs quite frequently	No resistance has ever been reported over number of treated generations	[88, 89]
Antibiofilm activity	Not very effective against biofilms	Effective antibiofilm agents with limited penetration	Relatively effective antibiofilm agents with higher destruction of biofilms	[13, 83]
Immune response	Generally non-immunogenic	Interaction with immune systems and susceptible to clearance by antibodies	Immunogenic, lower degree of antibody neutralization	[26, 51, 90]
Pharmacokinetics	Establish the relationship between concentration and the magnitude of killing activity	Little clinical evidence that defines optimal dosages and pharmacokinetic parameters of therapy	Defined concentration at site of infection and in blood circulation	[29, 88, 89]

*CPP* cell-penetrating peptide

### Endolysin therapy for G^+^ bacterial infections

Increasing interest in endolysins comes with emerging bacterial resistance and increasing need for novel antimicrobial agents. Given the natural structure of exposed bacterial cell wall without outer membrane barriers, endolysin therapy usually works best against infections caused by G^+^ bacteria [93]. Immediate lysis occurs without the need of holins or other partner enzymes when applied exogenously. Therefore, extensive experimental studies have focused on pathogenic G^+^ bacteria since the discovery of endolysins, especially after treatment failures increased considerably in *S*. *aureus*, *Streptococcus* sp., *Enterococcus* sp., and *B*. *anthracis* infections treated with antibiotic alone [17, 94, 95]. Most in vivo studies still concentrate on evaluating the efficacy of newly identified endolysins in the treatment of systemic infections, pneumonia, and nasal and skin infections caused by G^+^ bacteria; the majority of these studies involved mouse models and targeted MRSA and streptococcal species. Thus, this section mainly focused on the endolysins (including chimeolysins) in pre-clinical and clinical trial phases. Table 3 summarizes the different endolysins targeting G^+^ bacteria, and it will be discussed further.

**Table 3:** Endolysins and related derivatives active against G^+^ bacteria

Endolysin and/or derivative	Phage	Antimicrobial spectrum	Enzymatic activity	Type of infection treated in vivo	Clinical trial phase	Clinical trials identifier/accession No	References
SAL200	SAP-1	*S.* *aureus*	Amidase and endopeptidase	Bacteremia	IIa	NCT03089697	[61]
Exebacase (CF-301 or PlySs2)	Prophage of *S.* *suis*	*S.* *aureus,* *Streptococcus*	Peptidase	Bacteremia	III	NCT04160468	[28, 62]
P128	CHAP domain (TAME phage K) + SH3b (lysostaphin)	*S.* *aureus*	CHAP	Bacteremia	II	NCT01746654	[96–98]
Staphefekt SA.100	M23 endopeptidase (lysostaphin) + Amidase (Ply2638) + SH3b (Ply2638)	*S.* *aureus*	Amidase and endopeptidase	Atopic dermatitis	I/II	NCT02840955	[64, 99]
Medolysin®	–	*S.* *aureus*	–	Bacterial wound infections	–	–	[100]
XZ.700	Staphefekt SA.100 deleted 44 amino acids region deleted	*S.* *aureus*	Amidase and endopeptidase	Skin infection	Pre-clinical	–	[64]
MV-L	MR11	*S.* *aureus*, *S.* *simulans*	Amidase and endopeptidase	Nares infection, sepsis	Pre-clinical	BAF33253	[81]
LysP108	P108	*S.* *aureus*	Amidase	Subcutaneous abscess	Pre-clinical	YP_009099525	[17]
80α	phi80α	*S.* *aureus*	Amidase and endopeptidase	Systemic infection	Pre-clinical	ABF71642	[101]
phi11	phi11	*S.* *aureus*	Amidase and endopeptidase	Systemic infection	Pre-clinical	YP_500516	[101]
LysK	K	*S.* *aureus*	Amidase and endopeptidase	Systemic infection	Pre-clinical	YP_024461	[101]
Ply2638	2638A	*S.* *aureus*	Amidase and endopeptidase	Systemic infection	Pre-clinical	AAX90995	[101]
Twort	Twort	*S.* *aureus*	Amidase and endopeptidase	Systemic infection	Pre-clinical	AAX92311	[101]
phiSH2	phiSH2 prophage	*S.* *aureus*	Amidase and endopeptidase	Systemic infection	Pre-clinical	BAE05642	[101]
LysWMY	phiWMY	*S.* *aureus*	Amidase and endopeptidase	Systemic infection	Pre-clinical	BAD83402	[101]
CHAP_K_	K	*S.* *aureus*	Endopeptidase	Nasal infection	Pre-clinical	4CT3_D	[18, 102]
ClyF	CD domain (Ply187) + CBD domain (PlySs2)	*S.* *aureus*	CHAP	Bacteremia and burn wound infection	Pre-clinical	-	[22]
LysSS	SS3e	*S.* *aureus*, *Salmonella*, *Escherichia* *coli*	–	Systemic infection	Pre-clinical	AAW51228	[103]
Ply6A3	PD-6A3	*Acinetobacter* *baumannii*, *E.* *coli*, *S.* *aureus*	–	Sepsis	Pre-clinical	ALM01856	[104]
gp144	ΦKZ	*Pseudomonas* *aeruginosa*, *S.* *aureus*, *E.* *coli*, *B.* *cereus*	Transglycosylase	–	–	AAL83045	[105]
LysGH15	GH15	*Staphylococcus*	Amidase and CHAP	Bacteremia	Pre-clinical	ADG26756	[26, 67, 106, 107]
PlyGRCS	GRCS	*S.* *aureus*, *S.* *epidermidis*	Endopeptidase	–	–	AHJ10590	[19]
ClyH	CD domain (Ply187) + non-SH3b (phiNM3)	*S.* *aureus*	Amidase	Intraperitoneal infection	Pre-clinical	–	[108]
MR-10	MR-10	*S.* *aureus*	–	Subcutaneous	Pre-clinical	–	[109, 110]
LysH5	PhiH5	*S.* *aureus*, *S.* *epidermidis*	Amidase and endopeptidase	–	–	ACE77796	[59, 68]
ClyS	CD domain (Twort) + CBD domain (phiNM3)	*S.* *aureus*	Endopeptidase	Intraperitoneal, nasal and skin infection	Pre-clinical	–	[73, 78]
ClyC	CD domain (Ply187) + CBD domain (LysSA97)	*S.* *aureus*	–	Bacteremia	Pre-clinical	–	[38]
Lys16	P68	*S.* *aureus*	CHAP	–	–	AAO83890	[101, 111]
LysSAP33	SAP33	*S.* *aureus*	CHAP	–	–	QDH45454	[42]
Pal	Dp-1	*S.* *pneumoniae*	Amidase	Nasopharyngeal infection	Pre-clinical	O03979	[16]
Cpl-1	Cp1	*S.* *pneumoniae*	Muramidase	Endocarditis, bacteremia, pneumonia, meningitis	Pre-clinical	NP_044837	[69, 112–114]
Cpl-7	CP-7	*S.* *pneumoniae,* *S.* *pyogenes,* *E.* *faecalis*	Muramidase	Embryo infection	Pre-clinical	P19385	[115, 116]
Cpl-711	CD domain (Cpl-7) + CBD domain (Cpl-1)	*S.* *pneumoniae*	Muramidase	Bacteraemia	Pre-clinical	–	[117]
PL3	CD domain (Cpl-7) + CBD domain (LytA)	*S.* *pneumoniae*	Amidase	Embryo infection	Pre-clinical	–	[118]
ClyJ	CD domain (PlyC) + CBD domain (SPSL1)	*S.* *pneumoniae*	CHAP	Bacteraemia	Pre-clinical	–	[119]
ClyJ-3	ClyJ variant	*S.* *pneumoniae*	CHAP	Bacteraemia	Pre-clinical	–	[120]
ClyJ-3 m	ClyJ-3 variant	*S.* *pneumoniae*	CHAP	Bacteraemia	Pre-clinical	–	[121]
23TH_48	23TH	*S.* *pneumoniae*	Amidase	–	–	QOI69927	[122]
MSlys	MS1	*S.* *pneumoniae*	Amidase	–	–	AQY55407	[123]
PlyC	C1	*S.* *pyogenes*	Amidase	Mucosal epithelium infection	Pre-clinical	AAP42310	[76]
PlyPy	MGAS5005 prophage	*S.* *pyogenes*	Endopeptidase	Bacteremia	Pre-clinical	AAM79913	[124]
PlyGBS	NCTC11261	Group B streptococci, *S.* *agalactiae*	Endopeptidase and muramidase	Vaginal and oropharynx infection	Pre-clinical	AAR99416	[74, 125]
PlySK1249	SK1249 prophage	*S.* *agalactiae*, *Streptococcus* *dysgalactiae*	Amidase and endopeptidase	Bacteremia	Pre-clinical	EGL49245	[126]
PlyG	γ-phage	*B.* *anthracis*, *B.* *cereus*	Amidase	Intraperitoneal infection	Pre-clinical	PFW40491	[24]
PlyPH	*B.* *anthracis* BA2805 genome	*B.* *anthracis,* *B.* *cereus*	–	Intraperitoneal infection	Pre-clinical	WP_098639153	[127]
LysPBC2	PBC2	*B.* *cereus*	Amidase	–	–	AKQ08512	[128]
PlyV12	Φ1	*E.* *faecalis*, *E.* *faecium*	Amidase	–	–	YP_009814814	[70]
LysEFm5	IME-EFm5	*E.* *faecium*	Amidase	–	–	YP_009200901	[129]
LysIME-EF1	IME-EF1	*E.* *faecalis*	Endopeptidase	Intraperitoneal infection	Pre-clinical	YP_009042672	[126]
LysEF-P10	EF-P10	*E.* *faecalis*	Endopeptidase	Balance of the gut microbiota	Pre-clinical	AQT27695	[130]
Ply3626	phi3626	*C.* *perfringens*	Amidase	–	–	NP_612849	[131]
Psa	St13	*C.* *perfringens*	Amidase	–	–	WP_011010276	[132]

–, unknown or data not available

#### Endolysin therapy against staphylococcal infections

*S.* *aureus* is a representative of G^+^ bacteria that can cause skin and soft tissue infections, fetal pneumonia, pericarditis, brain abscess, bacteremia, and toxic shock syndrome [2, 133, 134]. Statistically, more than 10% of bloodstream *S.* *aureus* infections are caused by MRSA in 15 European countries, and the resistance rates are closer to 50% in some of these countries [3]. MRSA is a superbug that can cause various infections on the human body and is often acquired in the hospital. These nosocomial infections have been acquired by infected individuals, and they are often difficult to treat.

In the case of antibiotic treatment failure, endolysin is an effective option to control MRSA infections [93]. For example, the recombinant endolysin MV-L can rapidly and completely lyse MRSA within 15 min in vitro and efficiently protect mice from intranasal and intraperitoneal challenge with MRSA [81]. Similarly, a chimeric endolysin ClyS efficiently lysed MRSA, vancomycin-intermediate *S.* *aureus*, and MSSA strains by > 2-log10 in vitro and protected against death caused by MRSA in mouse nasal decolonization and bacteremia models [73]. Eight endolysins (80α, phi11, LysK, P68, 2638A, Twort, phiSH2, and LysWMY) display varied lytic activities against numerous staphylococcal strains in vitro, including cell surface mutants, drug-resistant strains, and their static biofilms. In a mouse model of systemic MRSA infection, these endolysins provide therapeutic potential and show no clinical symptoms at the end of treatment [61]. The above research results indicated that endolysin is highly effective in combating refractory infections caused by drug-resistant *S.* *aureus*.

Furthermore, several endolysins for the treatment of *S.* *aureus* infections are close to clinical application. P128 is an engineered endolysin (chimer) developed by the Indian company Gangagen, and it is currently in the clinical development stage. P128 is active against globally prevalent drug-resistant clinical *S.* *aureus* and *S.* *epidermidis* isolates. It exerts potent activity against sinus-derived *S.* *aureus* biofilms and is developed for clearing *S.* *aureus* nasal colonization and MRSA infection in mice and dogs [97, 98, 135, 136]. Staphefekt SA.100 and XDR.300 are commercially available recombinant endolysins that have been applied to patients with chronic skin infections caused by *S.* *aureus* [137]. Exebacase (also termed CF-301 or PlySs2) is considered an attractive agent that has rapid bacteriolytic activity, biofilm elimination capacity, and anti-staphylococcal potentials ranging from bacteremia to osteomyelitis when combined with other antibiotics. In addition to the above properties, exebacase has a minimal propensity for resistance development, no cross-resistance with antibiotics, and delayed post-antibiotic effect in vitro and in vivo [138]. Another endolysin, CHAP_K_, has the potential to reduce *S.* *aureus* colonization in the skin; thus, it may be used as a disinfecting agent in the healthcare environment [13]. Overall, endolysin treatment is a promising approach for staphylococcal infections.

#### Endolysin therapy against streptococcal infections

*S.* *pneumoniae* is another clinically important G^+^ bacterium that can cause different diseases ranging from a streptococcal pharyngitis to life-threatening pneumonia [77]. Endolysins have been successfully determined in a mouse model with streptococci. Cpl-1 and Pal alone or in combination have been used in the treatment of pneumococcal infections [80, 139]. A single dose of aerosolized Cpl-1 can rescue mice from fatal pneumococcal pneumonia [140]. Cpl-1 can reduce intranasal *S.* *pneumoniae* and ultimately prevent the development of acute otitis media following infection with influenza virus [141]. A single intracisternal injection of Cpl-1 (20 mg/kg) and intraperitoneal administration of Cpl-1 (200 mg/kg) decreased pneumococci in cerebrospinal fluid by 3-log10 and 2-log10, respectively, representing a promising alternative treatment option for pneumococcal meningitis [113]. Cpl-1 also shows therapeutic effects on *S.* *pneumoniae* rat endocarditis [112] and murine pneumococcal bacteremia [69]. In a mouse model of nasopharyngeal colonization, Pal was found to reduce *S.* *pneumoniae* to undetectable titers (log_10_ 0 CFU/10 mL nasal wash) 5 h after a single dose treatment and did not induce Pal-resistant pneumococci after extensive exposure to the enzyme [16]. Therefore, Cpl-1 and Pal are potent antimicrobial agents for the prevention and treatment of mucosal and systemic pneumococcal infections. Moreover, PlySs2 derived from a *S.* *suis* phage has broad lytic activity against group A *Streptococcus*, group B *Streptococcus*, group G *Streptococcus*, group E *Streptococcus*, and *S.* *pneumoniae*. PlySs2 (128 μg/mL) led to a 3-log reduction in the growth of *S.* *pyogenes* within 1 h, and 2 mg of PlySs2 protected 92% (22/24) of mice from bacteremia caused by mixed MRSA and *S.* *pyogenes* infections [82]. Similar results were observed with endolysin PlyC and PlyGBS [23, 76, 125]. These promising results have led to great research interest in the treatment of streptococcal infections with endolysins.

#### ***Endolysin therapy for infections caused by other G***^+^***bacteria***

Among G^+^ bacteria, *Staphylococcus* and *Streptococcus* are the most common in clinical settings. Therefore, many studies on endolysin-related treatment have been conducted, whereas research on endolysin therapy of other G^+^ bacteria is relatively lacking. However, an increasing number of studies on endolysin treatment of other G^+^ bacteria have been performed in recent years. For example, PlyV12 from an *E.* *faecalis* phage and LysEFm5 from an *E.* *faecium* phage have been described to be useful in the treatment of mucosal infections [70, 129]. *B.* *cereus* and *B.* *anthracis* are pathogenic bacilli that can cause serious harm to their hosts via food poisoning and anthrax toxicity, respectively [142]. Two recombinant endolysins PlyG and PlyPH are effective therapeutic agents for the control of *B.* *cereus* and *B.* *anthracis* both in vitro and in vivo [24, 127]. Moreover, endolysins have been recommended as impressive agents against drug-resistant pathogen *C.* *perfringens*, which can cause the infection of over 95% of chickens [13]. The endolysins Ply3626 and Psm are expected to be applied to poultry with broad lytic activity against *C.* *perfringens* [13, 131]. Interestingly, LysZ5 shows excellent activity against *L.* *monocytogenes* in soya milk and is greatly needed in food safety and food processing systems [13]. Therefore, these studies demonstrated that endolysins may be used to either eliminate or reduce G^+^ bacterial colonization from mucosal epithelium of either carriers or infected individuals and systemic infections, paving the way for endolysins to be applied as alternatives to the treatment of associated diseases in humans and animals.

#### *Synergistic effects of endolysins to combat G*^+^*bacteria*

In the treatment of G^+^ bacterial infections, the antimicrobial spectrum and antibacterial capacity of endolysins can be improved through the synergistic effect between the endolysins and antibiotics or other enzymes with diverse enzymatic specificities [17, 62, 143, 144]. Staphylococcal lysostaphin and LysK were found to exhibit strong synergistic activity on the MRSA strain USA300 and the mastitis-causing strain *S.* *aureus* 305 through checkerboard assay [145]. Two anti-pneumococcal endolysins Cpl-1 and Pal with muraminidase and amidase activities, respectively, were synergized in vitro and exhibited increased activity compared with either individual endolysin in a mouse pneumococcal infection model [80, 139, 146].

The synergistic effect between different endolysins or other cell wall hydrolases (i.e., lysostaphin) can be explained by the enhanced destructive effect generated when two different bonds were simultaneously cleaved within the 3D peptidoglycan meshwork. Alternatively, the cleavage of the first bond by one enzyme can result in better accessibility to the second target site by the other endolysin, causing a faster degradation of the substrate. A novel chimeric endolysin ClyS exhibited a typical pattern of synergistic action with both vancomycin and oxacillin in vitro. More importantly, ClyS and oxacillin at low doses that were no protective individually were found to present synergistic effects against MRSA septic death in a mouse model [73]. The combination of a staphylococcal endolysin Lys11 and an antimicrobial peptide R8K can enhance the bacteriolytic action against *S.* *aureus*, including MRSA clinical strains [147]. Animal experiments suggested that the synergistic antibacterial effects of LysP108 and vancomycin greatly reduce the area of subcutaneous abscess of mice infected with MRSA [11]. Concurrently, combinations of endolysins with antibiotics not only increase bactericidal efficacy but also resensitize drug-resistant bacteria, such as Cpl-1 and penicillin, MV-L and vancomycin, and SAL200 and standard-of-care antibiotics, such as nafcillin and vancomycin [81, 148, 149]. Therefore, the optimal combination of endolysin and other antimicrobial agents can help control the development of bacterial resistance and reduce the required antibiotic dosage [13, 150, 151].

### Safety of endolysins

As for applications, endolysin safety is an inescapable issue. Numerous experimental studies have shown that endolysins are innocuous after both topical and systemic administration in mice [51, 69, 76]. Treatment with endolysin specific to Group A streptococci does not bring about any histopathologic abnormalities in the mucosa and skin tissues of mice when administered daily for 7 days [51]. SAL200 is an endolysin-based candidate against *S.* *aureus*; it shows no toxicity and adverse effects in mice, dogs, and monkeys under pre-clinical safety evaluation [29, 152, 153]. Furthermore, SAL200 was found to be well tolerated among healthy male volunteers in a human single dose-escalating (0.1–10 mg/kg) study. More than three participants had some adverse effects, such as fatigue, stiffness, headache, and myalgia; most adverse effects were transient, mild, and self-limiting [29]. A high-dose intravenous injection of LysGH15 (10 mg) did not induce remarkable side effects (after 10 days) or pathological changes in the tissues of mice infected with *S.* *aureus* [26]. No severe allergic reactions as adverse events were found in pre-clinical studies, such as SAL200 and CF-301 [77, 152, 154]. Eukaryotic cells did not have peptidoglycan; thus, endolysins are expected to be safe in humans [52]. However, the main concern with respect to the safety of endolysins is the release of pro-inflammatory factors and bacterial components (e.g., lipopolysaccharide) during bacteriolysis, which may be directly toxic for eukaryotic cells [155]. To date, the side effects of endolysins have not been reported, thereby strongly supporting the safety of endolysin-based drug treatments.

### Future challenges and possible solutions

Given the global prevalence of multidrug-resistant bacterial infections, endolysins are considered as an attractive therapeutic option in clinical settings. Although endolysins show many advantages in the treatment of drug-resistant bacteria (Table 2), some challenges remain, and further research must be performed to consider their large-scale production, engineering, and drug delivery toward widespread utilization.

The large-scale production of phage endolysins has attracted great attention. However, the two main challenges that need to be solved are manufacturing cost and safety. *Escherichia* *coli* is the most common organism for the production of recombinant proteins, as this expression platform is well-established, and the cellular and molecular tools needed in the process of protein expression from gene cloning to protein purification are widely accessible [156, 157]. In general, *S.* *aureus* recombinant endolysins are expressed in *E*. *coli* [157]. However, some functional recombinant proteins are unavailable due to protein toxicity to the host or aggregation in inclusion bodies, even if numerous studies have attempted to optimize the *E*. *coli* expression system in the aspects of host engineering, expression vector design, and culture optimization [156]. Alternatively, the *Pichia* *pastoris* expression system has high recombinant protein yields; even some filamentous fungi or other systems can be considered for the production of recombinant endolysins [157, 158]. For example, the endolysins Cpl-1 and Pal, which are specific against *S*. *pneumoniae*, can be successfully expressed in chloroplasts of *Chlamydomonas* *reinhardtii*; this host has many advantages, such as the lack of endotoxins, no infectious potential, and low production costs [159]. Moreover, a platform for expressing an endolysin against *Cutibacterium* *acnes* in cyanobacteria can reduce production costs and avoid toxicity issues caused by toxic bacterial components such as endotoxin [157].

Furthermore, new strategies are needed to develop novel and suitable endolysins that possess enhanced bactericidal activity; expanded lytic spectrum; and increased solubility, stability, and circulating half-life. The modifications of native endolysins by molecular engineering and specific designing can completely create new enzymes with several improved features. Several approaches have been applied to modify endolysin enzymes, including domain deletion, addition, shuffling, and site-directed modifications. Virion-associated lysins (VALs) are another class of phage-encoded enzymes with antimicrobial activities that have been engineered to act on certain bacteria by fusing them into a chimeric lysin. EC300 and P128 are good examples of the VAL-derived chimeolysins that efficiently target *E*. *faecalis* and *S*. *aureus*, respectively [97, 160]. This breakthrough facilitates the generation of novel customized proteins that use not only VAL domains but also other agents, such as antimicrobial peptides, bacteriocins, and bacteriolysins [32]. A chimeric protein K-L, composed of the CHAP endopeptidase and amidase domains of LysK, as well as the glycyl-glycine endopeptidase domain of lysostaphin, displays increased stability in the presence of block copolymers of poly-L-glutamic acid and polyethylene glycol. The chimeric design of K-L reduces the immunogenicity of the enzyme [161]. A study demonstrated that the addition of cysteine to the C-terminal (CTC modification strategy) of antimicrobial peptide or lysin can increase the efficacy against both G^+^ and G^−^ pathogens by at least twofold [161]. Perhaps the suitable formulation of therapeutic compounds containing various endolysins, such as phage cocktail therapy, can enhance the lytic activity against specific bacteria, extend lytic spectrum, and decrease the chance of bacterial resistance.

**Fig. 4:**
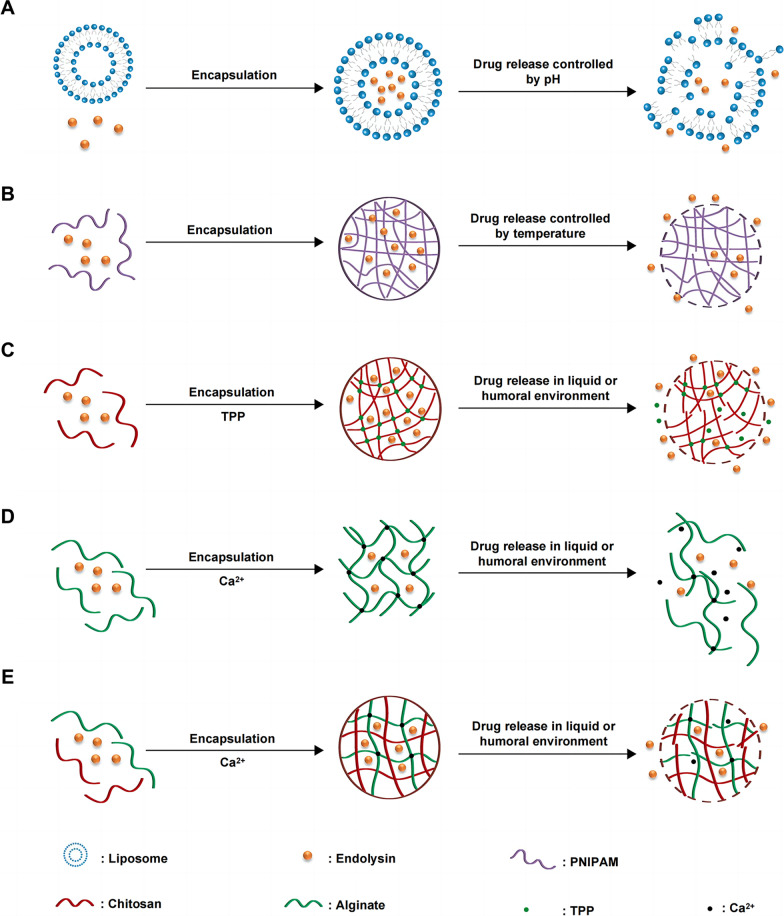
The delivery strategies for endolysins. Endolysins can be encapsulated in pH-sensitive liposomes (**A**), thermally triggered PNIPAM nanoparticles (**B**), chitosan nanoparticles (**C**), alginate hydrogel (**D**), and alginate-chitosan hydrogel (**E**). These nanoparticles and hydrogels can release endolysins at infection sites triggered by different circumstances and displayed lytic activity both in vitro and in vivo. *PNIPAM* poly N-isopropylacrylamide, *TPP* sodium tripolyphosphate

Finally, a considerable hurdle to the application of endolysin therapy is drug delivery. Many endolysins are used for topical treatment, such as skin bacterial infections, whereas the systemic application of endolysins remains challenging. In oral administration, endolysins can easily be degraded by stomach acids and proteases, which lead to poor bioavailability and irreversible damage to the integrity of the protein structure [93]. The encapsulation technique has offered a novel way of protecting endolysins until they reach their desired targets; this approach may enable unsuitable endolysins to become effective therapeutic agents. The release of the endolysin-encapsulated nanoparticles can be triggered by different environmental conditions (e.g., temperature or pH) or certain host- or pathogen-produced stimuli (e.g., cytokine, enzymes, secreted toxins, or signaling molecules) [137, 162]. Some successful results regarding the encapsulation of endolysins have been reported. The encapsulation of LysRODI endolysin in pH-sensitive liposomes can reduce planktonic *S*. *aureus* and its biofilm at pH 5 (Fig. 4A) [163]. The endolysin CHAPk and lysostaphin encapsulated in the thermally triggered poly(*N*-isopropylacrylamide) (PNIPAM) nanoparticles can be released in the *S.* *aureus* infection sites at 37 °C (Fig. 4B) [164]. Cpl-1-loaded chitosan nanoparticles are promising biocompatible candidates with increased bioavailability and *in-vivo* half-life for the treatment of *S.* *pneumoniae* infections (Fig. 4C) [25]. Moreover, chimeric ClyC-loaded alginate hydrogel (ClyC-AH) can retain the stability and activity of ClyC, decrease cytotoxicity, and reduce bacterial burden in a mouse *S*. *aureus* osteomyelitis model (Fig. 4D) [165]. In addition, the alginate-chitosan hydrogel delivery system can efficiently transfer the anti-staphylococcal endolysin LysMR-5 in vivo (Fig. 4E). Compared with the blank alginate-chitosan hydrogel, LysMR-5-loaded hydrogel shows enhanced bactericidal activity and good biocompatibility [166]. Apart from the examples described above, there are several studies about endolysin delivery, such as nanoparticles of chitosan derivatized with diethylaminoethyl (DEAE) groups encapsulating the Cpl-711 pneumococcal chimeric lysin [167], liposomes loaded with the endolysin MSlys [168], and pH-responsive nanoparticles of self-assembling peptide fusion with the endolysin P128 [169]. With further exploration of endolysins, these challenges can be overcome in the near future, facilitating the clinical application of phage endolysins.

## Conclusion

The unique mode of action, the rapid killing activity against bacteria (including persisters), and the low probability of resistance development are appealing features of endolysins for their application as alternatives of antibacterial agents. Many studies have shown that endolysins are effective antimicrobial agents that have synergistic effects with diverse antibiotics and antimicrobial peptides. With the high priority for the development of novel agents against multidrug-resistant bacteria, phage endolysins are promising candidates that serve as therapeutic options for controlling G^+^ bacterial infections. In the face of challenges such as activity, stability, cost, and ready-to-use drug availability in endolysin therapy, engineering modification (e.g., chimeric endolysins), production process optimization, and drug delivery development can be used to enhance the potential of endolysins, making endolysins a clinically proven drug to combat the crisis of drug-resistant bacteria in the future.

## Data Availability

Not applicable.
